# Comparison of basal cell carcinoma subtypes observed in preoperative biopsy and Mohs micrographic surgery^☆^^☆☆^

**DOI:** 10.1016/j.abd.2020.04.005

**Published:** 2020-06-27

**Authors:** Felipe Bochnia Cerci, Elisa Mayumi Kubo, Betina Werner

**Affiliations:** a Dermatology Service, Hospital de Clínicas, Universidade Federal do Paraná, Curitiba, PR, Brazil; b Postgraduate Program in Internal Medicine and Health Sciences, Universidade Federal do Paraná, Curitiba, PR, Brazil; c Department of Pathology, Hospital de Clínicas, Universidade Federal do Paraná, Curitiba, PR, Brazil

**Keywords:** Biopsy, Carcinoma, basal cell, Mohs surgery, Pathology

## Abstract

**Background:**

The treatment of basal cell carcinoma depends on its histological subtype. Therefore, a biopsy should be performed before definitive treatment. However, as the biopsy is only a sample of the tumor, it does not always shows every histological subtype present in the neoplasm. Few studies have compared the histological findings of biopsies with the findings of Mohs micrographic surgery. By evaluating the totality of the peripheral margins, in addition to sampling large tumor areas, this technique provides a more representative amount of tissue than preoperative biopsy.

**Objectives:**

a) Determine the agreement between the histological subtype of basal cell carcinoma from punch biopsy and the findings of Mohs surgery; b) To assess, among the discordant cases, the prevalence of non-aggressive tumors in the preoperative biopsy that were reclassified as aggressive by Mohs surgery.

**Methods:**

Retrospective analysis of 79 cases of basal cell carcinomas submitted to punch biopsy and subsequent Mohs surgery.

**Results:**

The agreement between the classification of the subtypes in the biopsy and in Mohs surgery was 40.5%. Punch biopsy was able to predict the most aggressive basal cell carcinoma growth pattern in 83% of cases.

**Study limitations:**

Retrospective nature, sample size, and biopsies performed by different professionals.

**Conclusions:**

The agreement between the histopathological subtypes of basal cell carcinoma as seen in preoperative biopsy and Mohs surgery was low. However, preoperative biopsy presented good accuracy (83%) in detecting aggressive histopathological subtypes.

## Introduction

Basal cell carcinoma (BCC) is the most prevalent malignancy in Brazil and worldwide, affecting mainly Caucasians.^1^ Although it rarely causes metastases, BCC can be locally destructive and is an important source of morbidity for patients, especially when located on the face.^2^ Thus, it must be adequately treated, and should not be disregarded, despite its slow growth. The treatment of BCC is based mainly on its histopathological subtype, location, and size.^3^, ^4^

BCCs can be histologically classified into six subtypes: superficial, nodular, micronodular, infiltrative, morpheaform, and metatypical.^5^, ^6^, ^7^ When more than one subtype is present in the same lesion, the BCC is classified as mixed.^8^, ^9^ In terms of aggressiveness, BCC can be divided into two groups: non-aggressive (superficial and nodular) and aggressive (micronodular, infiltrative, morpheaform, and metatypical).^6^ Aggressive BCCs have a higher risk of recurrence, especially when treated improperly.

In general, the treatment of choice for BCC is surgical excision.^3^, ^4^ However, superficial BCC in low-risk areas may also be treated by non-invasive modalities such as photodynamic therapy, imiquimod, or 5-fluorouracil, in addition to curettage and electrodissecation.^10^ For nodular or aggressive BCC, surgical excision is the most adopted treatment modality, with lateral margins of 4 mm and 6 mm, respectively. For BCCs located in cosmetically sensitive areas, mainly on the face, Mohs micrographic surgery (MMS) is the first line treatment.^10^, ^11^ In this technique, the assessment of all surgical margins occurs in the intraoperative period, allowing preservation of healthy tissue and leading to a higher cure rate.

In order to determine the best therapeutic option, it is essential to confirm the diagnosis and determine the histological subtype of the BCC through previous biopsy.^4^, ^10^, ^12^ However, preoperative biopsy represents only a sample of the tumor and may not encompass all histological characteristics of the whole lesion.^13^ Failure to detect aggressive subtypes can result in undertreatment and tumor recurrence. Therefore, it is important to determine the proportion of patients in whom the preoperative biopsy can identify the most aggressive histological subtype of BCC.

Therefore, this study aimed to:

a) assess the agreement of the histological subtype of BCCs in preoperative biopsies with those of MMS;
b) investigate, among the discordant cases, the prevalence of non-aggressive tumors in preoperative biopsy that were reclassified as aggressive during MMS.

## Methods

This was a retrospective study of a consecutive sample of patients operated by the same dermatologist using the MMS technique at a private clinic and at the Dermatology Service of Hospital de Clínicas, Universidade Federal do Paraná, from August 2016 to December 2018.^14^ The study was approved by the Human Research Ethics Committee (57268416.1.0000.0100).

Inclusion criteria were patients older than 18 years who underwent MMS for treatment of BCC previously biopsied by 3 or 4 mm punch. Only cases in which it was possible to assess the preoperative biopsy slide were included. These cases were selected from two private dermatopathology laboratories and from the Pathological Anatomy Laboratory at Hospital de Clínicas, Universidade Federal do Paraná. Cases which had a preoperative biopsy performed at other laboratories were excluded, due to the difficulty in accessing histopathological slides, as the biopsy had often been performed in other cities. Patients whose preoperative biopsy was performed using a method other than punch biopsy and patients whose tumor was not identified at MMS were also excluded. This can occur when the margins were clear in the first stage and debulking was not performed, or when no tumor was observed in the examined histological sections.

The demographic data and other data related to the tumor and surgery were extracted from the authors’ database (Microsoft Excel®), in which the data are routinely entered by the author, immediately after each surgery. The extracted data were gender, age, Fitzpatrick phototype, tumor location, largest tumor diameter, primary or recurrent tumor, and number of stages in MMS.

**Figure 1:**
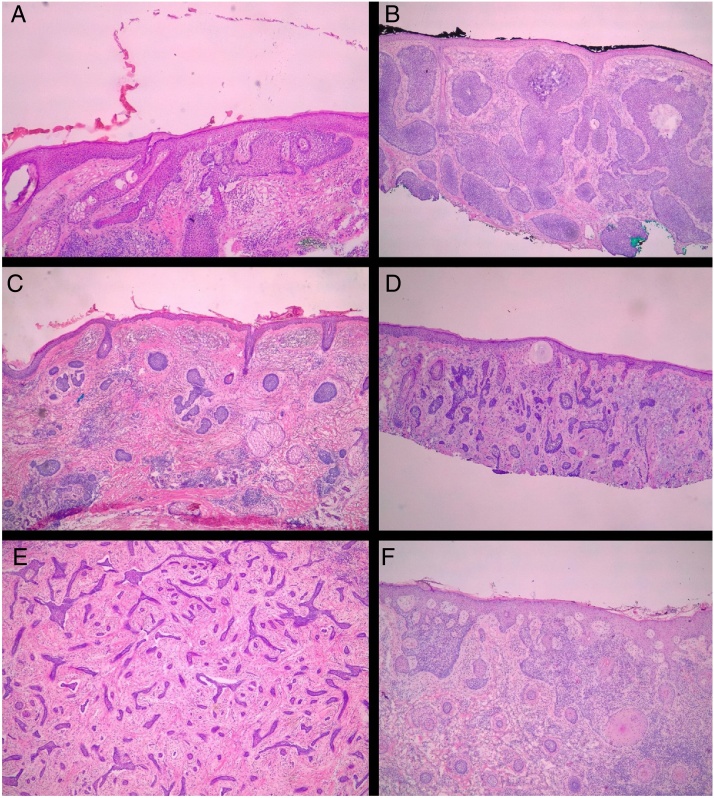
Histological subtypes of basal cell carcinoma. (A) Superficial; (B) Nodular; (C) Micronodular; (D) Infiltrative; (E) Morpheaform; (F) Metatypical. (Hematoxylin & eosin, ×40).

All pre-MMS biopsies were blindly reviewed by the study authors (BW and FBC), without knowledge of the histological result observed in MMS.^14^ All specimens obtained at MMS were blindly reviewed by the Mohs surgeon, without the knowledge of the histological type observed in the biopsy. BCCs were classified according to Sexton et al., stratifying the histopathological subtypes of BCCs into superficial, nodular, micronodular, infiltrative, morpheaform, metatypical, or mixed^7^ (Fig. 1). The superficial and nodular subtypes were grouped as non-aggressive, while micronodular, infiltrative, morpheaform, and metatypical BCCs were classified as aggressive.

After comparing the preoperative biopsy with the findings of MMS, the cases were classified as discordant when the histopathological subtypes present were not identical. The presence of any area of a given histological subtype was considered for the assessment.

After the evaluation of histopathological subtypes in both preoperative biopsy and MMS, the cases were divided into three groups: (1) the group of cases in which MMS showed a less aggressive subtype than that shown in the biopsy (downstaging); (2) the group of cases in which histological aggressiveness was higher in MMS when compared to biopsy (upstaging); and (3) the group of cases in which there was no change in the degree of aggressiveness between the biopsy and MMS classification (similar).^14^

The data obtained were digitized in an Excel™ table (version 14.0.6023.1000, Microsoft Office Professional Plus 2010©, Microsoft Corporation) and analyzed using SPSS™ software (v. 22.0, IBM).

Initially, a descriptive analysis of the data set was performed. Subsequently, for distribution analyses, Komolgorov–Smirnov normality tests were applied for quantitative variables, as well as non-parametric Mann–Whitney tests. When the criterion variable was categorical, the chi-squared test and Fisher's exact test were applied.

In all statistical tests, a 5% significance level was adopted.

## Results

Of the 258 cases of MMS operated by the author (FBC) in the period, 79 BCCs from 70 patients (29 men, 41 women) were included. Nine patients had two BCCs. The mean age was 64 years (36–93 years). The majority (*n* = 36) were classified as Fitzpatrick phototype III, followed by Fitzpatrick II (*n* = 33), and one patient, as Fitzpatrick IV.^14^

**Figure 2:**
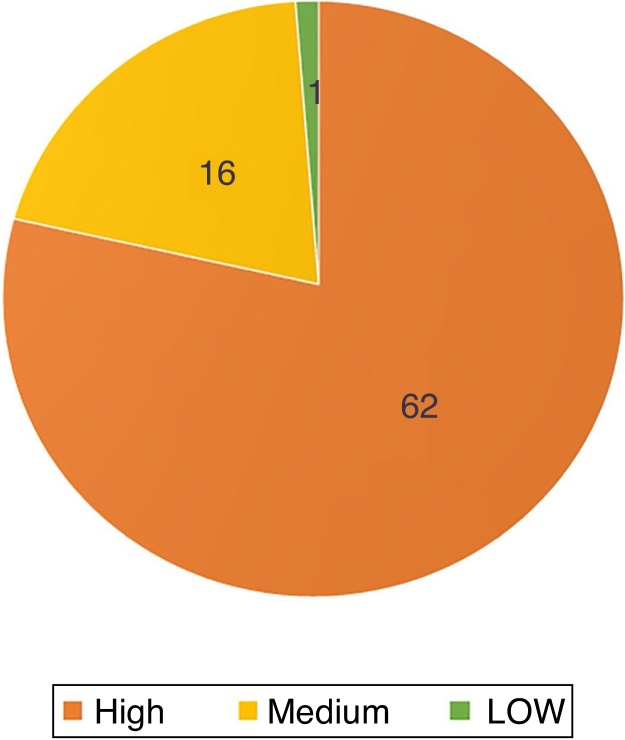
Location of tumors based on risk areas.

Regarding the topography of the tumors, except for one located on the chest, all were located in the cephalic and cervical region (*n* = 78; 98.7%). Sixty-two (78.5%) affected high-risk areas, with 44 on the nose (56% of the total; Fig. 2).

The mean of the longest axis of the tumor was 10.7 mm (3–40 mm), 37 (47%) of which were larger than or equal to 10 mm. The mean size of non-aggressive BCCs was 9.9 mm (3–19 mm), while that of aggressive tumors was 10.9 mm (3–40 mm). When classified as mixed or single subtype, the mean size was 11.1 mm and 9.6 mm, respectively.

Seventy-five (95%) were primary and four (5%) were recurrent. Among these, two had been submitted to conventional surgery, one to cryosurgery and the other to radiotherapy. The mean number of stages required for complete tumor removal was 1.56 (1–5).

## Histopathological evaluation of preoperative biopsy

**Figure 3:**
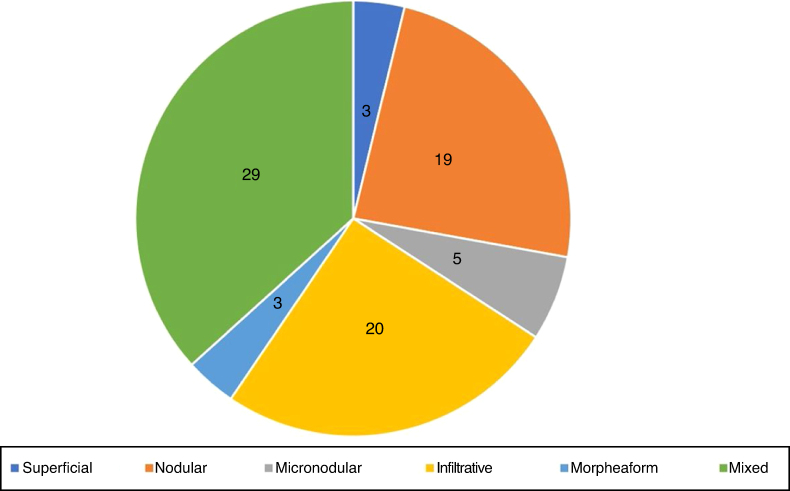
Histopathological subtypes at the preoperative biopsy.

Regarding the histopathological classification, in the punch biopsy, 25% (*n* = 20) of the BCCs were infiltrative, 24% nodular (*n* = 19), 6% micronodular (*n* = 5), 4% (*n* = 3) superficial, 4% (*n* = 3) morpheaform, and 37% (*n* = 29) mixed (Fig. 3). Of these, 23 were composed of two subtypes, five of three subtypes, and one of four subtypes. The nodular subtype was observed in 86% (25/29) of mixed BCCs. The most frequent combination was nodular with infiltrative (*n* = 9). Regarding aggressiveness, 62% (*n* = 49) were considered as aggressive and 38% (*n* = 30) as non-aggressive. The presence of any aggressive component was sufficient for inclusion in this group. In 27% (21/79), the presence of an aggressive and non-aggressive histopathological subtype was observed in the same lesion.

## Histopathological evaluation at MMS

**Figure 4:**
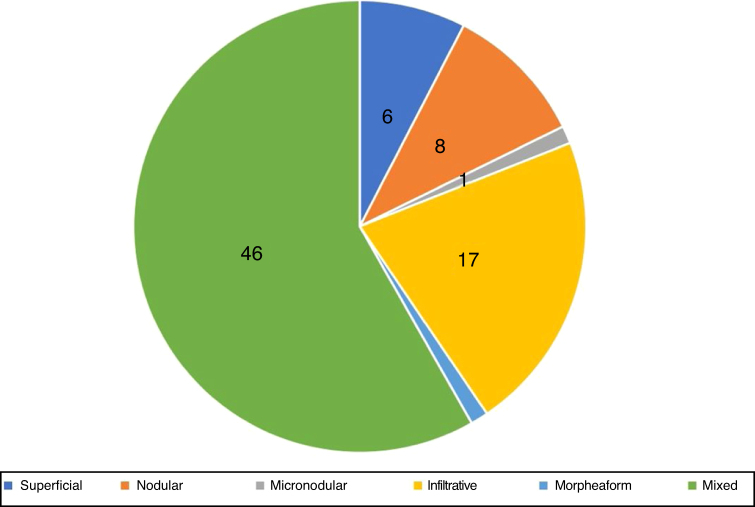
Histopathological subtypes at Mohs micrographic surgery.

**Figure 5:**
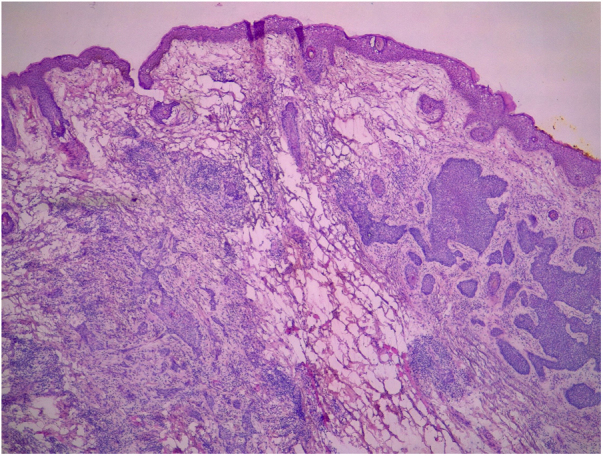
Mixed basal cell carcinoma composed of nodular and infiltrative subtypes. (Hematoxylin & eosin, ×10).

In the histopathological classification of MMS, 22% of BCCs were infiltrative (*n* = 17), 10% were nodular (*n* = 8), 1% micronodular (*n* = 1), 1% (*n* = 1) morpheaform, 8% (*n* = 6) superficial, and 58% (*n* = 46) mixed (Fig. 4). Of these, 24 were composed of two subtypes and 12, of three subtypes. The nodular subtype was present in 80% (37/46) of mixed BCCs. The most frequent combination was nodular with infiltrative (*n* = 11; Fig. 5). Regarding aggressiveness, 29% (*n* = 23) were non-aggressive and 71% (*n* = 56) were aggressive. In 43% (*n* = 34/79), the presence of an aggressive and non-aggressive histopathological subtype was observed in the same lesion.^14^

## Mixed BCCs

The total number of mixed BCCs, taking into account the combined findings of punch biopsy and MMS, was 57 (72%). The number of aggressive BCCs was 61 (77%).

## Agreement of histological subtypes

**Figure 6:**
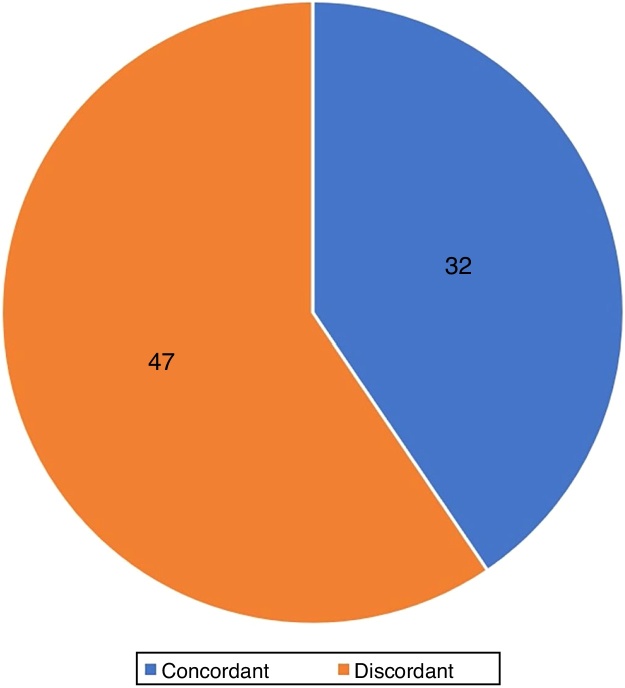
Agreement between histopathological subtypes at preoperative biopsy and Mohs micrographic surgery.

The agreement between the histological subtypes observed in the punch sample and in the MMS was 40.5% (32/79) (Fig. 6).

## Accuracy of biopsy to detect histological aggressiveness

**Figure 7:**
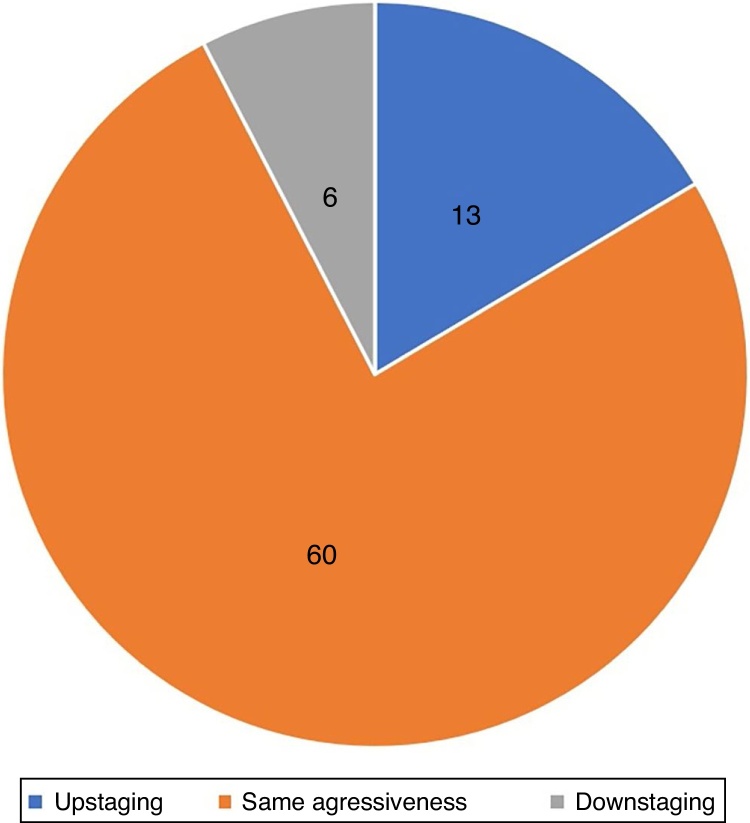
Aggressiveness agreement at preoperative biopsy and Mohs micrographic surgery.

Preoperative biopsy evidenced the most aggressive component of the tumor in 83% of cases. The prevalence of non-aggressive tumors in the preoperative biopsy that were reclassified as aggressive during MMS was 17% (*n* = 13; upstaging). In 7% (*n* = 6), downstaging was observed, in which preoperative biopsy showed an aggressive subtype that was not observed at MMS. In 76% (*n* = 60) of the tumors, the aggressiveness observed in the preoperative biopsy and in MMS was the same (Fig. 7).

When the 57 mixed BCCs were analyzed, the most aggressive component of the tumor was not detected in the preoperative biopsy in 23% (13/57) of the cases.

## Agreement of tumor aggressiveness between the Mohs surgeon and the dermatopathologist

Given the inherent subjectivity of any histopathological evaluation, an agreement test was performed regarding the aggressiveness of the tumors in the preoperative biopsy, which was substantial (kappa = 0.610).

## Correlation of the number of stages of MMS *vs.* tumor aggressiveness in preoperative biopsy and at MMS

When only the histopathological findings from the preoperative biopsy were considered, non-aggressive tumors had a mean of 1.26 stages in MMS, while aggressive tumors presented a mean of 1.73 stages (*p* = 0.025). When only the histopathological findings of MMS were considered, non-aggressive tumors had a mean of 1.48 stages in MMS, while aggressive tumors presented a mean of 1.59 stages (*p* = 0.81).

In the study sample, no difference was observed between “change in aggressiveness status” and tumor size or location (areas of high, medium, and low risk; *p* = 0.955). No difference was observed between “change in aggressiveness status” and primary or recurrent BCC (*p* = 0.305).

## Discussion

Three previous studies compared the agreement of the histopathological subtypes of the preoperative biopsy with that observed at MMS. Izikson et al. observed an agreement of 49%; Stiegel, 46.7%; Orengo, 42.7%; and the present study, 40.5%.^15^, ^16^, ^17^ The rigorous classification of the histopathological subtypes adopted in the current study may have influenced the lower agreement. The presence of any distinct subtype area, even if minimal, was considered. Another important observation is that Stiegel did not debulk the tumors at MMS for additional evaluation, thus reducing the amount of tissue that was compared with the preoperative biopsy.^16^ This measure probably increased the possibility of agreement of the findings. Genders et al. conducted the only study in which the MMS block was completely sectioned and was not debulked, which allowed the observation of the largest possible tumor sample.^18^ In daily practice, the block is not completely sectioned, as this increases the cost and, mainly, surgical time. Furthermore, the presence of some deep cuts representing 100% of the peripheral margin is sufficient to consider the margins free. It is important to note that debulking is not mandatory, since its portion is not part of the margin to be assessed. Debulking is one of the techniques used in MMS to facilitate flattening of the lateral margins so that they are in the same plane as the deep margin, allowing the longitudinal cuts to include 100% of the surgical margin.

In studies that compared the histological subtypes of the preoperative biopsy with wide local excision (WLE), the agreement ranged from 54% to 87%.^8^, ^19^, ^20^, ^21^, ^22^, ^23^ When compared with studies with MMS, this greater agreement is possibly due to the fact that in MMS more tissue is evaluated, increasing the probability that small areas with other subtypes are identified (and thus reducing the agreement).

The discrepancy rates between the subtypes observed in the preoperative biopsy and in the WLE or MMS can be explained by some factors, such as variation in the preoperative biopsy method (punch or shaving), size of the biopsy, choice of the tumor area to be biopsied, types of BCC studied (primary or recurrent), and the criteria used for the classification of subtypes (which vary according to the reference). For example, Russell et al. used a simplified classification that stratified BCCs into superficial, nodular, and infiltrative.^22^ In addition, the rigor used in the classification directly interferes with the number of mixed BCCs, which varied significantly between studies (from 18% to 74%), being 72% (57/79) in the present study.^6^, ^24^, ^25^

From a practical standpoint, the accuracy in detecting the most aggressive subtype in the preoperative biopsy is more important than the accuracy in diagnosing the histopathological subtype, as the treatment of BCC is strongly based on whether it is histologically aggressive. The presence of an aggressive subtype that is not detected in the biopsy may be responsible for the failure of certain treatments and a higher recurrence rate.^26^

**Figure 8:**
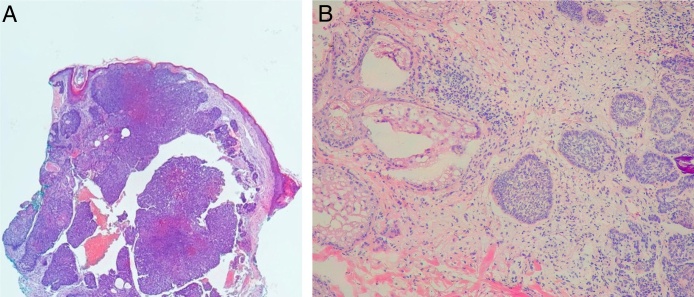
Upstaging. (A) Preoperative biopsy: nodular basal cell carcinoma. (B) Mohs surgery: micronodular basal cell carcinoma. (Hematoxylin & eosin,×10 and ×40, respectively).

In the present study, the accuracy of preoperative biopsy in detecting the aggressive subtype of BCC was 83%.^14^ That is, in 17% of the cases, the biopsy did not detect the most aggressive subtype (Fig. 8). Previous studies that made this same analysis, comparing preoperative biopsy with MMS findings, found values of 19%, 27%, and 36%.^15^, ^16^, ^17^ In a recent review on the subject that included studies with WLE and MMS, Singh found an upstaging rate of 31%.^27^ Possible explanations for the lower rate of upstaging in the present study are the fact that, in the United States, where the other studies were performed, the insurance coverage for MMS is much broader, which is why non-aggressive tumors are frequently treated with the technique. This is reflected in the number of aggressive BCCs (in the preoperative biopsy) in Singh's review (24.5%) when compared to the present study (62%).^27^ This directly interferes with the chance of upstaging, since a greater number of aggressive tumors in the preoperative biopsy reduces the chance of progression to aggressive tumors in MMS.

The high discrepancy in the study by Stiegel et al. (36%) can be partly explained by the methodology used by those authors. Superficial BCCs that demonstrated a nodular component in MMS were also considered as upstaging.^27^ In other studies, this change was not considered as upstaging, because superficial BCC and nodular BCC were considered non-aggressive.

The biopsy method may also have influenced the results. The study by Stiegel included cases in which the preoperative biopsy was performed by punch or shaving methods. Being superficial, shaving biopsy may fail to identify subtypes of BCC located deeper in the dermis.^17^ In Stiegel's study, among the tumors that were reclassified as more aggressive, 84% had been sampled by shaving biopsies and 14%, punch biopsy (the others were not reported).^16^ However, in a previous study, Haws observed only a slight superiority of punch biopsy over shaving in the correct detection of BCC subtypes (89% *vs.* 81%).^8^

Regarding studies that compared preoperative biopsy with WLE, the upstaging rate was lower, ranging from 7% to 26%.^8^, ^9^, ^20^, ^21^, ^22^, ^23^, ^24^ The adoption of different criteria between studies to determine the agreement of preoperative biopsy with MMS or WLE affected this discrepancy. When compared with MMS studies, the lower rate of upstaging can partly be justified by the fact that in MMS more tissue is evaluated than in WLE, with a greater chance of disagreement regarding aggressiveness as well.

In the present sample, in 61% of the cases that presented upstaging, the preoperative biopsy indicated nodular BCC, but failed to detect the aggressive subtypes of the tumor (infiltrative or micronodular), which is similar to that reported by Kamyab et al. (63%).^21^ Among BCCs initially classified as nodular on preoperative biopsy, 42% (8/19) demonstrated an aggressive subtype in MMS, a finding superior to that reported by Wolberink et al. (17%).^20^

The aggressiveness observed in the preoperative biopsy was able to better predict the number of stages in MMS than the aggressiveness observed in the MMS itself. Such data had not been evaluated in previous, similar studies. When only the histopathological findings from the preoperative biopsy were considered, non-aggressive tumors had a mean of 1.26 stages in MMS, while aggressive tumors presented a mean of 1.73 stages (*p* = 0.025). When only the histopathological findings of MMS were considered, non-aggressive tumors had a mean of 1.48 stages in MMS, while aggressive tumors presented a mean of 1.59 stages (*p* = 0.81).^14^

What distinguishes the present study is that the histological classification of the BCCs was blind. In most other studies, data were collected from a database, or the assessment was not blind.^9^, ^16^ When assessing the WLE or MMS slides, a non-blinded evaluator may be influenced by the result of the initial biopsy.

Traditionally, the Mohs surgeon assesses histological slides during the procedure.^28^ For this, these surgeons undergo intense training in dermatopathology of skin tumors. In the United States, for example, Mohs surgeons perform about 1.500 MMSs during fellowship, over one year.^29^ Studies carried out in different countries have demonstrated a 99% degree of agreement between Mohs surgeon and dermatopathologist in the evaluation of clear margins.^30^, ^31^, ^32^, ^33^ In the present study, the agreement of the aggressiveness of the BCCs was assessed in the preoperative biopsy; the kappa score demonstrated a substantial agreement between the Mohs surgeon and the dermatopathologist. This variation in the classification of subtypes has already been evaluated in other studies, such as that by Genders et al., in which two dermatopathologists evaluated the MMS slides, with substantial agreement, similar to the present study.^18^

Another aspect evaluated was downstaging, in which the preoperative biopsy showed an aggressive subtype that was not observed in MMS. It occurred in 8% (*n* = 6) of the cases in the present study, *vs.* 40% in the study by Genders and 17% in the study by Stiegel.^16^, ^18^ In three cases, the infiltrative subtype observed in the preoperative biopsy (along with nodular or superficial) was not seen in MMS, but only the nodular or superficial. There are two possible justifications for downstaging. Insufficient histological sections of a nodular BCC located deep or tangential to the sectionning plane may reveal only small tumor “nests.” These small tumor clusters may be incorrectly identified as micronodular BCC on preoperative punch biopsy. Another possibility is that a histological subtype that was present in the biopsy tissue was removed by the biopsy itself, not appearing in the definitive excision.^21^

In 76% of the tumors (*n* = 60), aggressiveness was the same in the preoperative biopsy and MMS, a finding consistent with most other studies.^16^, ^18^, ^27^

The mean size of the tumors on its longest axis was 10.7 mm; this data is seldom available in other studies.^15^, ^16^, ^34^ However, as in the studies by Genders and Wolberink, size did not influence the change in tumor aggressiveness.^18^, ^20^ Moreover, recurrent tumors (despite the small sample size hindering comparison) and location did not influence changes in aggressiveness.

The fact that the biopsies were performed by different physicians is a limitation, as it influences the choice of the biopsy site. Other limitations were the limited sample size and the retrospective nature of the study. However, the latter was minimized, since the data are prospectively entered into the authors’ database. Finally, a relative limitation (since this is not routine in the technique) is the fact that the blocks were not sectioned entirely at MMS. This reduces the amount of tissue that is examined. However, the evaluation of debulking and peripheral margins allows the evaluation of a greater amount of tissue than in WLE.

## Conclusions

The present study demonstrated that the agreement between the histopathological subtypes of the BCCs seen in the preoperative biopsy and in the MMS was low (40%). However, preoperative biopsy presented good accuracy (83%) in detecting aggressive histopathological subtypes. Considering that the treatment of BCC is based on the most aggressive subtype observed in the preoperative biopsy, the chance of undertreatment is present in approximately one in six cases of BCC.

Variables such as location, size, and recurrent tumors were not correlated with the change in aggressiveness observed between preoperative biopsy and MMS.

Dermatologists should be aware of the limitation of preoperative biopsy in the diagnosis of aggressive subtypes of BCC.

## Financial support

None declared.

## Authors’ contributions

Felipe Bochnia Cerci: Approval of the final version of the manuscript; conception, and planning of the study; elaboration and writing of the manuscript; obtaining, analyzing, and interpreting the data; intellectual participation in propaedeutic and/or therapeutic conduct of studied cases; critical review of the literature.

Elisa Mayumi Kubo: Conception and planning of the study; obtaining, analyzing, and interpreting the data.

Betina Werner: Approval of the final version of the manuscript; obtaining, analyzing, and interpreting the data; effective participation in research orientation; critical review of the manuscript.

## Conflicts of interest

None declared.
